# Clinical, Epidemiological, and Mycological Characteristics of Dermatophyte Infections: A Cross-Sectional Study From a Tertiary Care Hospital

**DOI:** 10.7759/cureus.104680

**Published:** 2026-03-04

**Authors:** Priyanka Kowe, Smita Ghate, Rachita Dhurat

**Affiliations:** 1 Dermatology, All India Institute of Medical Sciences, Nagpur, Nagpur, IND; 2 Dermatology, Lokmanya Tilak Municipal Medical College and General Hospital, Sion Hospital, Mumbai, IND

**Keywords:** dermatophytosis, epidemiology, itraconazole treatment, tinea, trichophyton rubrum

## Abstract

Background: Dermatophyte infection is a prevalent infection of the skin, hair, and nails caused by a fungus of multiple genera. The distribution and frequency of dermatophytosis vary with the geographical region and various epidemiological factors.

Aim: This study aimed to describe the clinical-epidemiological and mycological profile of dermatophytosis and observed the response of systemic antifungals in cases of tinea corporis and tinea cruris.

Methodology: A descriptive cross-sectional study was carried out among 338 patients of clinically diagnosed cases of dermatophyte infection presented to the dermatology outpatient department in a tertiary care hospital between January 2014 and March 2015, considering inclusion and exclusion criteria. Sample collection for fungal elements was done from skin scraping, nail clippings, and hair plucks.

Results: Out of 338 patients included in the study, 212 (62.7%) were males, and 126 (37.3%) were females. The mean age of patients was 30.7 ± 14.5 years. The maximum number of patients (51.8%) was in the age group of 21-40 years. Maximum patients were laborers by occupation (28.7%) and belonged to the lower middle class (53.9%). The majority of 103 (30.47%) patients reported in July. The most common symptom was itching, noted in all 338 (100%) patients, followed by scaling in 294 (87%) of patients. Annular erythematous plaque with scaling (261 (76%)) was the most common clinical finding. Two hundred eighty-two (83.43%) patients reported sharing fomites, while 239 (70.71%) patients had contact with infected family members. Forty-two percent of patients were wearing tight-fitting clothes daily. Two hundred sixty-eight (79.29%) patients had a history of use of different topical medications in the past, and 64 (18.8%) patients reported a history of systemic antifungals taken in the past six months. Comorbid conditions were seen in 80 (23.67%) patients. One hundred twelve (33.13%) were overweight, 54 (15.9%) were obese, and 39 (11.53%) patients were underweight. Tinea corporis was the most common clinical pattern seen in 100 patients (29.5%), followed by tinea cruris in 75 patients (22.3%). Direct microscopy with KOH was positive in 273 (80.76%) patients, whereas culture was positive in 160 (46. 74%) patients. *Trichophyton rubrum* was the predominant species, isolated in 97 patients (60.6%), followed by *Trichophyton mentagrophyte* in 58 patients (36.32%). Among the four antifungal drugs, persistence of disease was maximum with the fluconazole group, and mycological cure was seen predominantly in the itraconazole-treated group.

Conclusion: Young male patients of the lower-middle-class family having a positive family history of dermatophytosis were more prone to dermatophytic infections. Overall, these findings highlight the multifactorial epidemiology of dermatophytosis, reaffirm *T. rubrum* as the predominant pathogen, and support itraconazole as the most effective systemic therapy in achieving sustained mycological cure.

## Introduction

Dermatophytosis is the most common fungal infection of superficial skin caused by filamentous keratinophilic fungi. They produce extracellular enzymes (keratinase) that are capable of hydrolyzing keratin. Hence, these fungi that feed on keratin and infect the skin, hair, and nails are known as dermatophytes. These are commonly known as tinea or ringworm.

Based on conidial morphology and arrangement (asexual form of the organism), these fungi are grouped into three genera: *Trichophyton*, *Microsporum*, and *Epidermophyton*. Hot and humid tropical climates, such as in India, promote fungal infections. Age, sex, seasonal variation, racial factors, genetic factors, lifestyle, certain medical conditions (such as diabetes mellitus (DM) and hypothyroidism), contact with animals, and contact with affected family members are the factors involved in the pathogenesis of these infections [1]. The distribution and prevalence of various species of infective dermatophytes vary among different areas [1]. Common species of dermatophyte that cause infections are *Trichophyton mentagrophyte*, *Trichophyton rubrum*, *Epidermophyton floccosum*, *Trichophyton schoenleinii*, *Trichophyton violaceum*, and *Microsporum audouinii*. Based on the anatomic site, dermatophyte infections can be classified as tinea capitis (head), tinea corporis (body), tinea cruris (groin), tinea pedis (foot), tinea manuum (hand), tinea unguium (nail), and tinea barbae (beard). The variation in clinical presentation depends upon the size of the inoculum, site of the body infected, and immune status of the host [2]. It is found that 30% to 70% of patients remain asymptomatic hosts. Climate, social practices, migration, and host factors influence the epidemiology of dermatophytosis [1]. The two most important methods used to diagnose dermatophytosis are direct microscopy with KOH and isolation of specific species through culture. The commonly encountered dermatophyte infections are treated both by topical and systemic antifungal therapy, but nowadays, there is an increasing recurrence and persistence of these infections despite available treatment due to antifungal resistance. Data on the clinical, epidemiological, and mycological aspects of dermatophytosis are lacking in our area; hence, we surveyed these parameters of dermatophytes. We also studied the mycological aspect and observed the response to four antifungal drugs in cases of tinea corporis and tinea cruris.

## Materials and methods

This was a descriptive cross-sectional study carried out among suspected cases of dermatophytosis (diagnosed clinically based on a round, annular, or polycyclic lesion with central clearing and raised erythematous, scaly, advancing border associated with itching) attending an outpatient department of a tertiary care hospital in Western India (Lokmanya Tilak Municipal Medical College and General Hospital, Mumbai, India) [3]. Patients of all ages and both genders who gave consent for the study were included. The study was commenced after acquiring clearance from the Institutional Ethics Committee (IEC) and conducted between January 2014 and March 2015 for a period of one year.

Sample size

The sample size was calculated by using the formula $n = Z^{2}p q/d^{2}$, where p is the prevalence, q is 100 - p, and d is the absolute error. We have taken p as 69.5% [4], q will be 100 - 69.5 = 30.5, and d is a 5% error. The z-value was 1.96. Thus, we got a minimum sample size of 325.279. We have recruited 338 patients.

Data collection

A predesigned questionnaire (Appendix) was used to collect the data, which contained an epidemiological history of patients including age, gender, occupation, socioeconomic status, peak season of the disease, history of sharing of fomite, family history of similar illness, and type of clothing, and clinical data including chief complaints, clinical signs, history of previous treatment, comorbidities, built, and clinical examination were collected.

Socioeconomic status

Patients were categorized into upper, lower, upper-lower, upper-middle, and lower-middle classes as per the Kuppuswamy scale based on the education, occupation, and income of the family per month. Body mass index (BMI) was calculated as weight (kg)/height² (m²). Patients were grouped into underweight (BMI < 18.5), normal (18.5-24.99), overweight (BMI = 25-29.99), and obese (BMI ≥ 30).

Clinical diagnosis of dermatophytosis

**Figure 1:**
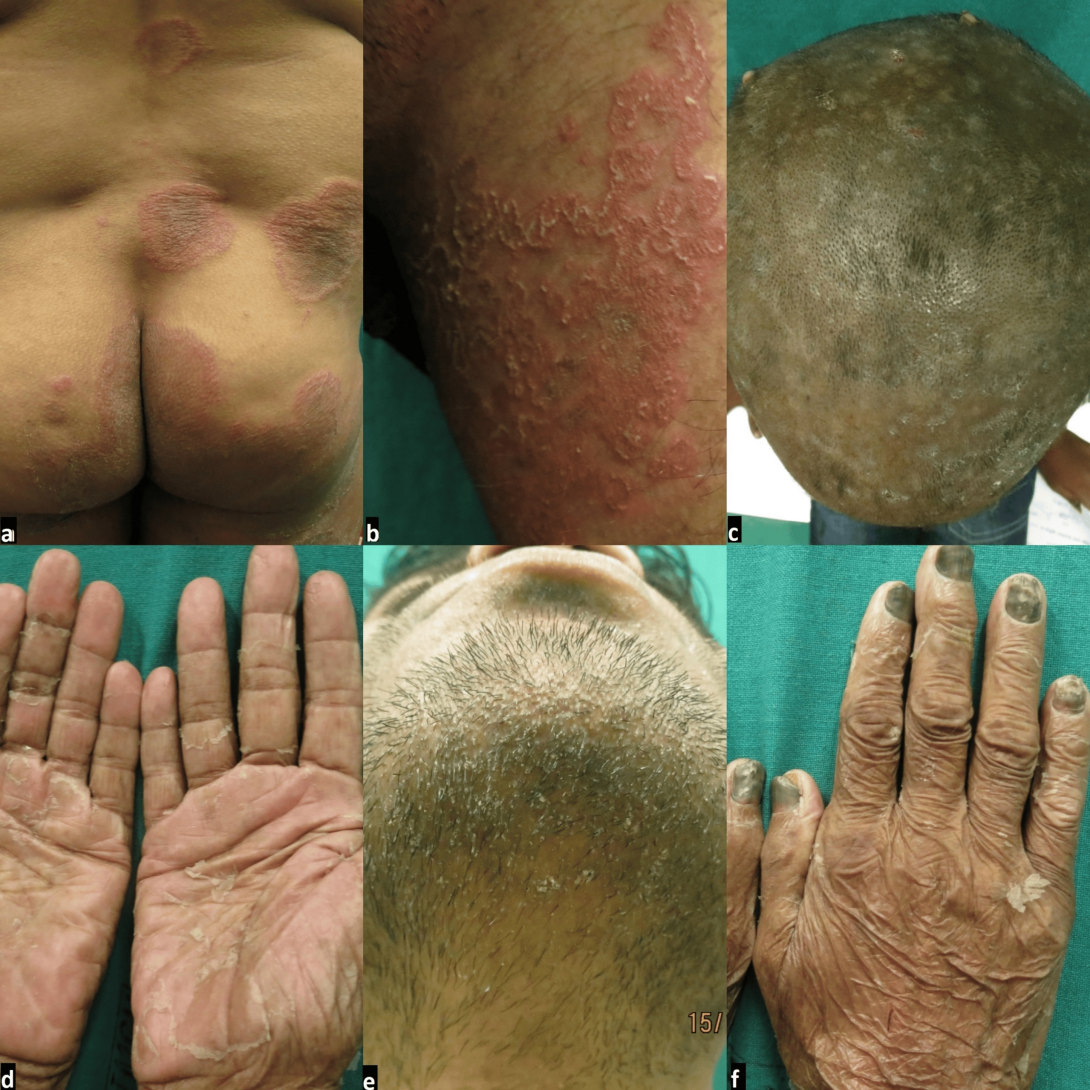
(a) Multiple erythematous scaly plaques with raised advancing border and central clearing over the back. (b) Multiple erythematous round plaques with red raised borders in the groin area. (c) Multiple gray-white scaly patches over the scalp. (d) Ill-defined scaly patches of palms. (e) Scaly, lusterless patches of the beard region. (f) Finger nail discoloration.

Clinically, tinea corporis was identified by dermatophytic involvement of glabrous skin, with exclusion of the palmar, plantar, and inguinal areas, with typical morphology such as annular or sometimes multiple plaques merging to form a plaque with polycyclic border with scaling over the entire active border (Figure 1a) [3]. The center of the plaque may exhibit scaling or complete clearance in the progressive lesion. Tinea cruris was diagnosed when there was the involvement of the groin, genitalia, pubic area, perineal, and perianal skin (Figure 1b). Tinea capitis was the dermatophytic infection of the hair and scalp, which was characterized by scaly gray patches associated with breakage of hair (Figure 1c). Some lesions showed boggy swelling studded with pustules, broken hair, and discharging pus. Tinea manuum and tinea pedis represented ringworm infection of palms and soles, respectively, with scaling and papulovesicular eruption (Figure 1d). Tinea barbae predominantly involved the beard area of men, which manifested as perifollicular papules and pustules with mild surrounding erythema, and hairs were lusterless, brittle, and easily pluckable (Figure 1e). Nail involvement by dermatophyte infection corresponded with tinea unguium with characteristic whitish to brownish-yellow opacities of the distal nail plate, subungual hyperkeratosis, leukonychia, and destruction of the nail plate (Figure 1f) [3].

Sample collection

**Figure 2:**
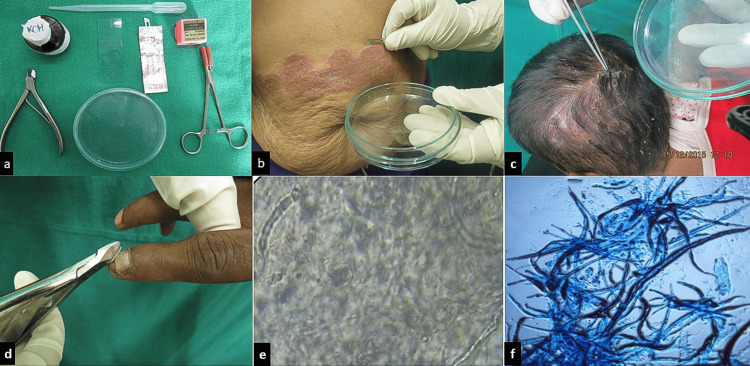
(a) Basic material required for sample collection, (b) skin scrapings from the margin of the erythematous plaque over the abdomen, (c) hair plucking in case of tinea capitis, (d) nail clippings for tinea unguium, (e) KOH wet mount showing long slender fungal hyphae, and (f) wet lactophenol cotton blue stain showing branched septate filamentous hyphae.

For skin scrapings, lesions were cleaned with 70% ethanol, and scrapings from the active border were collected using a sterile blunt scalpel (Figure 2). Samples were placed in sterile dishes and examined microscopically after 10% KOH treatment for 20 minutes. In suspicious cases, lactophenol cotton blue stain was used to confirm the diagnosis. In suspected tinea capitis or barbae, dull hairs were plucked with sterile forceps, ensuring inclusion of the basal root, the site of fungal concentration. For tinea unguium, affected nails were cleaned with alcohol and clipped; subungual debris was also collected when present. Nail samples were treated with 20% KOH overnight before microscopy. All specimens were labeled, sealed, and sent for culture on Sabouraud dextrose agar (SDA) with antibiotics and cycloheximide and incubated at 26°C for two weeks. For demographic analysis, all dermatophyte variants were included. To evaluate responses to four antifungals, only cases of tinea corporis and tinea cruris were selected. A total of 175 patients with tinea corporis/cruris received one of four systemic antifungals such as terbinafine (N = 84; 250 mg/day), fluconazole (N = 36; 150 mg/week), griseofulvin (N = 28; 250 mg twice daily), or itraconazole (N = 16; 100 mg twice daily) for 30 days and were followed up on days 30 and 60. Eleven patients were lost to follow-up; thus, 164 were analyzed. All antifungals were discontinued after one month, and day-60 follow-up was assessed for persistence or recurrence of lesions. KOH mount and culture were performed at follow-up to determine mycological cure. Patients having disease on follow-up day 30 and day 60 despite proper treatment in cases of tinea corporis and tinea cruris were labeled as having persistence of disease. Mycological cure was defined as negative KOH and culture.

## Results

Table 1 depicts the sociodemographic profile of the 338 patients included in the study. Males constituted the majority (62.7%), while females accounted for 37.3%. The age of patients ranged widely from two to 82 years, with a mean age of 30.7 ± 14.5 years. Most patients (51.8%) belonged to the 21-40-year age group, followed by 40-60 years (22.5%). The pediatric (1-12 years) and adolescent (13-19 years) groups comprised 11.2% and 10.9%, respectively, whereas the geriatric group represented only 3.6%. When pediatric and adolescent patients were combined, they accounted for 22.1% of cases, suggesting possible contact transmission within households and communities. Occupationally, laborers (28.7%) formed the largest group, followed by students (26.6%) and housewives (25.4%), with smaller proportions of tailors (5.6%), vendors (4.4%), and others (9.2%). Socioeconomic assessment using the Kuppuswamy scale showed that over half of patients (53.9%) belonged to the lower-middle class, with fewer in the upper-middle (16.6%), upper-lower (14.5%), lower (8.3%), and upper (6.5%) classes.

**Table 1:** Sociodemographic profile of the cases.

Demographic variables		No. of cases	Percentages (%)
Age groups in years	1-12	38	11.2
	13-19	37	10.9
	20-39	175	51.8
	40-60	76	22.5
	>60	12	3.6
Gender	Males	212	62.7
	Females	126	37.3
Occupation	Laborer	97	28.7
	Students	90	26.6
	Housewife	86	25.4
	Tailor	19	5.6
	Vendor	15	4.4
	Others	31	9.2
Socioeconomic status	Lower middle	182	53.9
	Upper middle	56	16.6
	Upper lower	49	14.5
	Lower	28	8.3
	Upper	22	6.5
Total		338	100

Peak season

**Figure 3:**
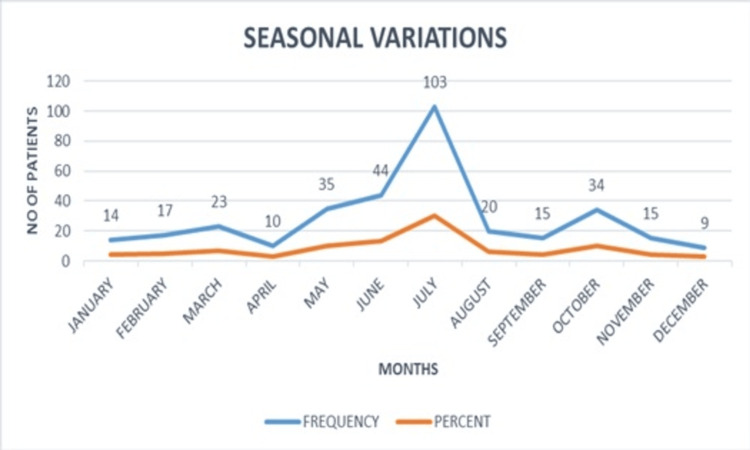
Seasonal variations of dermatophytosis, with the highest peak in the month of July.

The maximum number of patients enrolled in our study was during the month of July (103 patients, 30.47%), followed by June (44 patients, 13.01%), May (35 patients, 10.35%), and October (34 patients, 10.05%). The fewest patients were seen in the month of December (9 (2.66%)) (Figure 3).

Duration of the present disease

In this study, a short duration of tinea infection was seen in the majority of patients, where the duration was ≤1 month, 270 (80.7%). Fifty-eight (17.1%) patients had the disease for 45 days, 41 (12.1%) patients for two months, and five (1.47%) patients for three months.

Clinical presentation (symptoms and signs)

The patient manifested with various signs and symptoms, the most common being itching, present in 338 (100%) patients, followed by scaling in 294 (87%) patients. Among the cutaneous findings, the most common was an annular erythematous plaque with scaling seen in 261 patients (76%), followed by scaly macules and patches in 49 patients (14.5%), plaque with pustule in 24 patients (7.1%), and scaling with pustule in five patients (1.5%).

Sharing of fomites (contact with infected patients through fomites)

Of 338 patients included in the study, 282 patients gave a history of contact with shared fomites. Of these 282 patients, 86 (25.4%) shared towels, 48 (14%) cloths, and 132 (39.1%) bedding. Contact with animals was observed in 16 (4.7%) patients (Table 2).

**Table 2:** Epidemiological history of the cases of dermatophytosis.

Variables	No. of patients	Percentages (%)
Sharing of fomites	282	83.43
Family history of a similar illness	239	70.71
Previous treatment taken	268	79.29
Systemic antifungal in the past	64	18.8
Comorbidities	80	23.67
Overweight	112	38.05
Obese	54	15.9
Underweight	39	11.53

Family history

Of 338 patients, 239 (70.71%) have a history of contact with infected family members.

Type of clothing

The present study showed that the maximum number of patients (42%) wore tight-fitting clothes daily, 30% of patients wore tight-fitting clothes alternate days, 20% wore loose-fitting clothes, and 8% wore tight-fitting clothes once a week.

Previous treatment

Of 338 patients, 268 patients had a history of use of different topical medications in the past. Of these 268 patients, 98 (28.9%) used steroids in the past, 58 (17.15%) used ayurvedic medications, and 112 (33.12%) patients took the help of over-the-counter medications (details of which patients were unable to recollect).

Systemic antifungals in the past six months

A total of 64 (18.8%) patients gave a history of previous systemic antifungals taken in the past six months. Out of these, 23 (6.8%) patients were prescribed terbinafine, 39 patients received fluconazole, and two (0.6%) patients used itraconazole in the past for dermatophytic infections.

Associated medical condition

Morbid conditions were seen in 80 patients. Of these 80 patients, 37 (10. 3%) had DM, 12 (3.55%) had hypothyroidism, seven (2.07%) had eczema, and four (1.18%) had psoriasis. Eleven (3.25%) patients had pulmonary tuberculosis, eight (2.36%) patients were infected with human immunodeficiency virus (HIV), and one (0.3%) patient was immunosuppressed due to chemotherapy.

Built

In the present study, 132 (39.05%) patients were of normal build, 112 (33.13%) were overweight, 54 (15.9%) were obese, and 39 (11.53%) were underweight.

Clinical pattern

**Figure 4:**
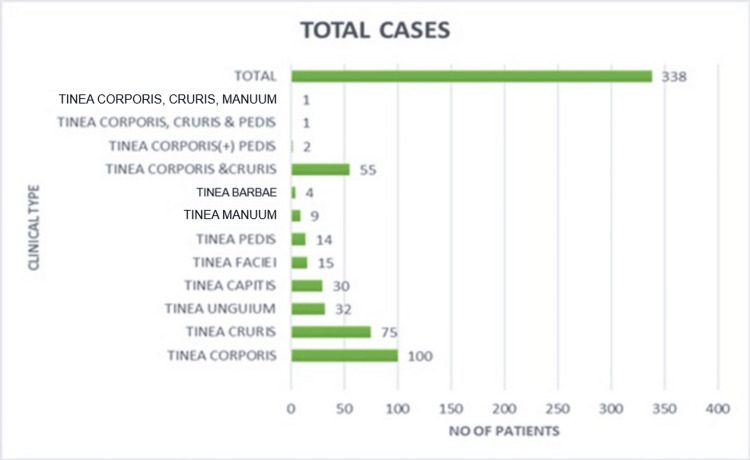
Clinical types of cases according to the site involved.

The most common clinical pattern among all dermatophytosis was tinea corporis (most frequent single-site infection), present in 100 patients (29.5%), followed by tinea cruris in 75 patients (22.3%), tinea unguium in 32 patients (9.4%), tinea capitis in 30 patients (8.8%), tinea faciei in 15 patients (4.4%), tinea pedis in 14 patients (4.1%), tinea manuum in nine patients (2.6%), and tinea barbae in four patients (1.1%). Fifty-eight patients had more than one site involved (body + groin in 55 patients = two clinical types; body + groin + feet in one patient = three clinical types; body + groin + hand in one patient) (Figure 4).

Microscopy

**Figure 5:**
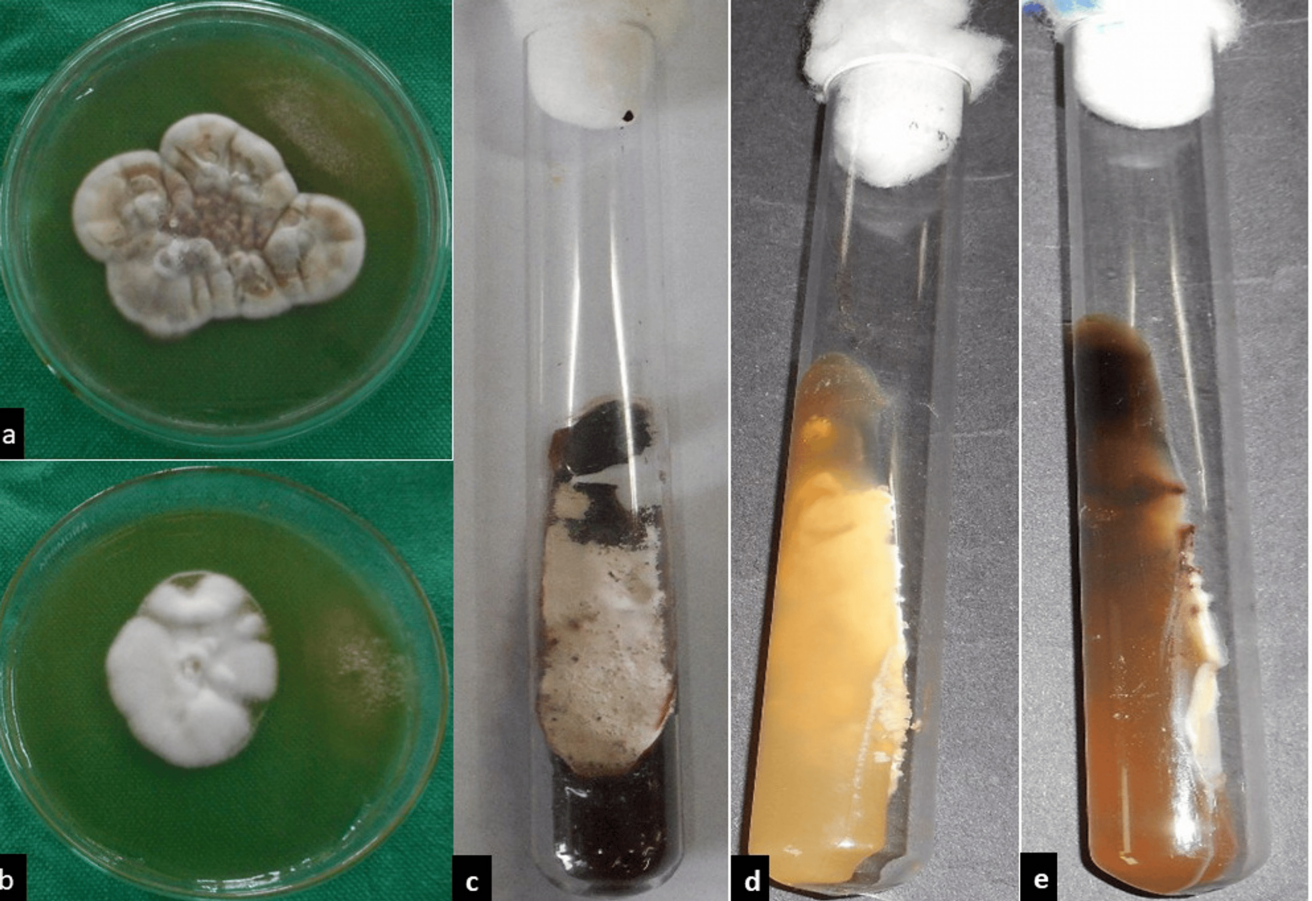
(a) Culture of Trichophyton rubrum: white, cottony colony; (b) Trichophyton mentagrophyte: pale, pink, fluffy, granular colony; (c) tube culture showing T. rubrum; (d) tube culture with T. mentagrophyte; (e) tube culture with Trichophyton violaceum.

Direct microscopy with KOH was positive in 273 (80.76%) patients, whereas culture was positive in 160 (46.74%) patients. *T. rubrum* was the predominant species, isolated in 97 patients (60.6%), followed by *T. mentagrophyte* in 58 patients (36.32%), *T. violaceum* in three patients (1.87%), and *M. audouinii* in two patients (1.25%) (Figure 5).

Out of the 16 patients of the itraconazole-treated group, three patients (18.8%) had persistence of disease; with terbinafine (n = 84), 30 patients (35.7%) showed persistence of disease; and with griseofulvin (n = 28), 11 patients (39.3%) had persistent disease at the end of two months. The maximum number of cases having persistence of disease was seen in the fluconazole-treated group (n = 36), with 28 (77.8%) patients. The association between them was found to be significant, as p < 0.05 (Table 3). Out of 16 patients treated with itraconazole, 14 patients (87.5%) had negative KOH smear and culture at the end of treatment; terbinafine showed 46 patients (54.7%) having KOH and culture negativity at the end of treatment. With griseofulvin, 15 patients (53.5%) had a negative report of KOH and culture post treatment. The fluconazole group showed negative KOH smear and culture in two patients (5.5%).

**Table 3:** Association between antifungal drugs and persistence of disease.

		Persistence of disease			Total
		No		Yes	
Fluconazole	n	8		28	36
	%	22.2%		77.8%	100.0%
Itraconazole	n	13		3	16
	%	81.3%		18.8%	100.0%
Terbinafine	n	54		30	84
	%	64.3%		35.7%	100.0%
Griseofulvin	n	17		11	28
	%	60.7%		39.3%	100.0%
Total	n	92		72	164
	%	56.1%		43.9%	100.0%
			Value	df	Asymp. Sig. (2-sided)
Pearson Chi square			23.413	3	3.31E - 05 (p < 0.05)
Likelihood ratio			24.310	3	2.15E - 05 (p < 0.05)

## Discussion

Among 338 dermatophytosis patients, this study demonstrated male predominance (62.71%). The ratio of 2:1 of males to females was also reflected in previous studies by Janardhan and Vani and Kalita et al. [5,6]. This was correlated with males having more outdoor physical activity, predisposing them to catch infections easily. Dermatophytosis affected individuals across all age groups; in this study, patients' ages ranged from two to 82 years. The highest prevalence was observed in the 20-39-year age group, i.e., young adults, accounting for 51.8% of the total population. This indicates that greater mobility and increased interpersonal contact likely contribute to the higher susceptibility in this group. Similar findings were observed by Janardhan and Vani, Kalita et al., and Poyyamozhi and Lakshmanan, with the 21-30-year age group constituting the most affected [5-7]. Most of the patients enrolled in our study were laborers (28.7%) by occupation, followed by students (26.6%), housewives (25.4%), tailors (5.6%), and vendors (4.38%). This finding was consistent with observations reported by Kalita et al. [6]. These findings were most likely to be due to increased physical activity in laborers and housewives and increased outdoor activities in students. All these factors lead to increased sweating, which predisposes to fungal infections. Dermatophytic infections were most common among the lower-middle class (53.91%) on the Kuppuswamy scale of socioeconomic status, and least common among the upper class (6.52%) in our study. Similar results were also seen in the study of Kalita et al. [6]. It may be attributed to overcrowding and poor hygienic conditions among people from lower socioeconomic strata. The most common season in which the maximum patients enrolled was July (30.47%), followed by June (13%), May (10.35%), and October (10.05%). This was in accordance with a previous study, which showed the most common season to be June-September (41.16%) [6]. It was also observed that the relative humidity of Western India was usually maximum in the months of June to September. From this, it can be concluded that high relative humidity predisposes to dermatophytic infections, which were also studied in the above-mentioned study. The comparison of demographic profiles in various studies is given in Table 4.

**Table 4:** Comparison of demographic and mycological profiles in various studies.

Studies	Age	Gender	Occupation	Socioeconomic status	Peak season	Most common type	Most common species	KOH positivity	Culture
Janardhan and Vani [5]	16-30 years (44%)	M > F (2:1)	Laborer (41%)	-	-	Tinea corporis	Trichophyton rubrum	90%	72%
Kalita et al. [6]	21-30 years (39%)	M > F (3:1)	Laborer (39%)	Lower middle	August-September	Tinea corporis	Trichophyton mentagrophyte	58.4%	44.07%
Poyyamozhi and Lakshmanan [7]	21-30 years (28%)	M > F	Laborer	Lower middle	-	Tinea corporis	T. rubrum	70%	41%
Present study	20-40 years (51.8%)	M > F (2:1)	Laborer (28.7%)	Lower middle (53.9%)	July-October	Tinea corporis	T. rubrum	80.76%	46.74%

In this study, a short duration of tinea infection was seen in the majority of patients having symptoms of ≤1 month (80.7%), which was comparable with a study by Dalei et al. [8]. The present study showed that 25.4% of patients shared towels, 14% clothes, and 39% beddings. A study done by Ayaya et al. showed similar findings in which 8.5% of patients shared towels, 2.6% shared clothes, and 18.8% shared beddings [9]. Similar findings were also seen in the study of Weitzman and Summerbell [10]. It was observed that 70.71% of patients had contact with infected family members who were suffering from dermatophytic infections. A comparable study done by Bindu and Pavithran showed a positive familial history suggestive of dermatophytosis in 16.6% of patients [11]. The pattern of clothing in our study showed that 42.01% of patients had a habit of wearing tight-fitting clothes daily, followed by tight-fitting clothes on alternate days (30.18%). These clothing pattern leads to occlusion and accumulation of sweat, which in turn predispose to dermatophytic infections. Similar findings were noticed in studies done by Poluri et al., where 40.32% of patients had a history of wearing tight and occlusive clothing [12]. Bindu and Pavithran also showed similar findings [11]. Two hundred sixty-eight patients had a history of use of different topical medications in the past. Use of steroids, ayurvedic medications, and over-the-counter medications was seen in 28.9%, 17.15%, and 33.12%, respectively, of the study population. These observations were also noted in the study of Agarwal et al. [13]. Dermatophytoses are associated with various medical conditions, which showed that 10.3% of patients were suffering from DM, which was also reported in the study of Poluri et al., where 8.06% patients were associated with diabetes, Sooriya et al. reported 16% as diabetics, and Rajamohanan et al. noticed 20.3% as diabetics [12,14,15]. Other associated factors observed were hypothyroidism in 3.55% of patients, eczema in 2.07%, and psoriasis in 1.18%. Kalita et al. showed an association of eczema and psoriasis in patients with dermatophytoses [6]. In our study, 3.2% of patients were immunocompromised due to tuberculosis and 2.36% due to HIV, and 0.3% were on chemotherapy. Association of HIV infection was studied in 13.83% of patients by Poluri et al. [12]. This association with HIV and hypothyroidism was also noticed in two patients of dermatophytoses in a study by Rajamohanan et al. [15]. In this study, 132 patients were of normal build (BMI = 18.5-24.9), 112 and 54 patients were overweight (BMI = 25-29.9) and obese (BMI ≥ 30), respectively, and 39 patients were underweight (BMI ≤ 18.5). This suggests that overweight and obesity are not contributing factors to the development of tinea infection. Tinea corporis was the most frequent diagnosis in the present study (29.5%), followed by tinea cruris, tinea unguium, tinea capitis, tinea faciei, tinea pedis, tinea manuum, and tinea barbae. The various combinations of infections seen were tinea corporis and tinea cruris in 55 patients; tinea corporis and tinea pedis in two patients; tinea corporis, tinea cruris, and tinea pedis in one patient; and tinea corporis, tinea cruris, and tinea manuum in one patient. These observations were consistent as reported by Janardhan and Vani and Kalita et al. [5,6]. In this study, direct microscopy with KOH wet mount examination was found to be positive in 80.76%, and positive culture of the fungus on SDA was 46.74%. These findings were comparable with those of Kalita et al. [6]. *T. rubrum* was the most common species identified on culture, being positive by 60.62%; this was followed by *T. mentagrophyte*, positive in 36.32%. A similar finding was noted by Janardhan and Vani [5]. The other isolates were *T. violaceum* (1.87%) and *M. audouinii* (1.25%). Mycological cure was found to be maximum (87.5%) with the itraconazole-treated group, followed by terbinafine (54.7%) and griseofulvin (53.5%). Mycological cure was least with fluconazole (5.5%). At two months, disease persistence was lowest with itraconazole (18.8%), followed by terbinafine (35.7%) and griseofulvin (39.3%). The highest persistence was observed with fluconazole (77.8%). The difference among treatment groups was found statistically significant (p < 0.05). Our findings of the lowest persistence with itraconazole and the highest with fluconazole were consistent with the findings of Singh et al. [16]. The mycological cure rate was maximum with itraconazole (87.5%), followed by terbinafine (54.7%), griseofulvin (53.5%), and fluconazole (5.5%). These observations were similar to studies by Singh et al. and Bhatia et al. [16,17].

Limitations

The relatively small sample size limited the detailed evaluation of epidemiological patterns and evolving trends. Larger multicentric studies are warranted for more robust insights. Moreover, the lack of antifungal susceptibility and resistance profiling restricted understanding of pathogen dynamics; future studies incorporating these aspects may better clarify dermatophytosis pathogenesis and treatment response.

## Conclusions

Dermatophyte infections are influenced by multiple epidemiological parameters, underscoring the importance of early preventive measures rooted in personal care and hygiene. To better understand the evolving clinical spectrum of dermatophytosis, future research should focus on large-scale studies that account for shifting epidemiological characteristics driven by environmental changes. *T. rubrum* was the chief pathogen. Among systemic therapies, itraconazole demonstrated superior mycological cure and lowest disease persistence, underscoring its effectiveness in current dermatophytosis management.

## Appendices

CASE RECORD FORM

Title: “Clinico-Epidemiological Study of Dermatophyte Infection in a Tertiary Care Hospital in Western India”

Sr No:

Name:

OPD/IPD:

Age/sex:

Registration number:

Occupation:

Address:

Mobile No:

Socioeconomic status: low/middle/high

Marital status:

CHIEF COMPLAINT:

(1) Itching

(a) Yes/no

(b) Duration

(c) Site

(2) Scaling

(a) Yes/no

(b) Duration

(c) Site

(3) Raised erythematous

(a) Yes/no

Border

(4) Hair loss

(a) Yes/no

(5) Nail changes

(a) Yes/no (thickening, discoloration, crumbling)

PAST HISTORY OF TINEA INFECTION:

(a) Yes/no. If yes,

- When:

- Treatment taken:

FAMILY HISTORY OF SIMILAR INFECTION:

(a) Yes/no. If yes,

- When:

- Treatment taken:

SHARING OF FOMITES AMONG FAMILY MEMBERS:

(a) Yes/no. If yes,

- Towel

- Clothing

- Comb

PERSONAL HISTORY:

Hygiene:

Bath:

(a) Every day

(b) Once every 2 days

(c) Twice a week

(d) Once a week

Clothing

(a) Every day

(b) Once every 2 days

Changes

(c) Twice a week

(d) Once a week

Type of clothing

(a) Tight-fitting/loose-fitting

Pets in the family

(a) Yes/no

Housing:

(a) Well-ventilated/ill-ventilated/crowded

Addiction

(a) Smoking

(b) Tobacco

(c) Alcohol

(d) Drugs

Workplace

(a) Sedentary

(b) Strenuous

HISTORY OF ANY MEDICAL ILLNESS:

(a) Diabetes/obesity/malnutrition

Yes/no. If yes,

- Duration:

- Treatment:

Other immunocompromised conditions: HIV/chemotherapeutic drugs/corticosteroids

History of any topical application: ayurvedic/homemade/homeopathic/OTC drugs/topical steroids

LABORATORY TESTS:

(1) Scraping and KOH preparation:

(2) Fungal culture:
